# The Relationship Between Perceived Uncontrollable Mortality Risk and Health Effort: Replication, Secondary Analysis, and Mini Meta-analysis

**DOI:** 10.1093/abm/kaad072

**Published:** 2024-01-08

**Authors:** Richard Brown, Gillian Pepper

**Affiliations:** Psychology Department, Northumbria University, Newcastle, UK; Psychology Department, Northumbria University, Newcastle, UK

**Keywords:** Health behaviors, Socioeconomic inequality, Uncontrollable mortality risk, Public health, Perceived control

## Abstract

**Background:**

The Uncontrollable Mortality Risk Hypothesis (UMRH) states that those who are more likely to die due to factors beyond their control should be less motivated to invest in preventative health behaviors. Greater levels of perceived uncontrollable mortality risk (PUMR) have been associated with lower health effort in previous research, but the topic remains understudied.

**Purpose:**

To examine the evidence for the UMRH by replicating a previous study investigating the effects of PUMR on social gradients in health effort, and conducting a mini meta-analysis of the overall relationship between PUMR and health effort.

**Methods:**

We replicated Pepper and Nettle (2014), who reported a negative relationship between PUMR and health effort, and that the positive effect of subjective socioeconomic position on health effort was explained away by PUMR. We also compared the predictive effect of PUMR on health effort with that of dimensions from the Multidimensional Health Locus of Control scale—a well-used measure of a similar construct, which is frequently found to be associated with health behavior. Finally, we conducted a mini meta-analysis of the relationship between PUMR and health effort from the available research.

**Results:**

PUMR was negatively associated with health effort, and mediated 24% of the total effect of subjective socioeconomic position on health effort, though this mediation effect was weaker than in Pepper and Nettle (2014). PUMR was shown to be a substantially stronger predictor of health effort than the relevant dimensions of the MHLC scale. Finally, our mini meta-analysis indicated a medium-sized negative relationship between PUMR and health effort.

**Conclusions:**

Our findings offer support for the role of PUMR in mediating the relationship between subjective socioeconomic position and health effort. The results highlight the importance of measuring and understanding PUMR in studying socioeconomic inequalities in health behaviors. We discuss potential areas for future research, including determining the accuracy of PUMR, investigating influential cues, examining the role of media in shaping risk perceptions, and understanding individuals’ awareness of their own perceptions of mortality risk.

## Teaser

People who are more likely to die from uncontrollable factors are less motivated to perform preventative health behaviours. Improving the safety of people’s living environments can have a positive impact on health behaviours.

## Introduction

This article explores the relationship between perceptions of uncontrollable mortality risk and health effort in the context of the Uncontrollable Mortality Risk Hypothesis (UMRH). We discuss the theoretical background and assumptions of the UMRH and describe how previous research has measured perceived uncontrollable mortality risk (PUMR) and its effect on self-reported health effort. We will set out the existing evidence of this relationship and highlight the need for replication and further research.

### The UMRH

The UMRH states that people who are more likely to die due to factors that are beyond their control should be less motivated to invest in preventative health behaviors [1]. Nettle [2] modeled the relationship between perceptions of control over risk and health behavior to provide the theoretical basis for the UMRH, leading the way for a behavioral ecological explanation of socioeconomic gradients in health behaviors.

Nettle [2] asserted that the optimal individual investment in health behavior should be less for people of lower socioeconomic status because they are typically exposed to greater levels of uncontrollable mortality risk. This suggests that humans have evolved psychological mechanisms that respond to environmental cues of risk to determine one’s level of investment in preventative health behaviors. Uncontrollable mortality risk refers to that portion of risk of death that cannot be mitigated by an individual allocating their energy to preventative health behavior. Meanwhile, controllable mortality risk is that which can be mitigated through the actions of the individual. Nettle [2] argued that the level of uncontrollable mortality risk within an environment should set a limit to the optimal amount of energy that is worth investing in preventative health behaviors. Although most sources of mortality risk cannot be considered wholly controllable or uncontrollable, there are varying degrees to which they may be deemed to be beyond individual control. For example, although some people are born with a greater disposition toward developing heart disease, individual cardiovascular risk is largely affected by individually controllable health behaviors (e.g., diet and exercise) [3, 4]. Similarly, adverse health effects from air pollution can be reduced by wearing masks, reducing exposure to pollutants in the home, or by avoiding heavily polluted areas [5, 6]. However, such actions will have limited efficacy and, if people are unable to change their environment, the overall risks to their health from air pollution over time will be largely uncontrollable.

Nettle’s model assumes that people in lower socioeconomic positions typically experience greater levels of uncontrollable mortality risk. This is consistent with a range of evidence showing a negative association between socioeconomic status and exposure to environmental risks that are harmful to human health; such as hazardous waste, air pollutants, inadequate housing, poor water quality, noise exposure, threat of violence, and COVID-19 infection and fatality rates [7–15]. The model assumes that, by allocating resources to investing in preventative health behaviors, there is a trade-off against other activities that may be beneficial to individuals within their environment. These competing activities might include developing social relationships, searching for a mate, accumulating resources, or increasing social status. Assuming a trade-off between investing in health and competing interests, the detrimental effects of lower socioeconomic position on health cause a reduction in the payoff from investing in preventative health behaviors. Because of this reduction in payoff, the optimal amount of investment in health is reduced for people in lower socioeconomic positions, which produces a secondary effect of lower investment in preventative health behaviors. Thus, the resulting inequality in health outcomes is worsened beyond the original environmental disparity to provoke a self-reinforcing feedback loop, which entrenches poverty. This suggests that lower levels of investment in health by socioeconomically deprived communities can be seen as the result of an adaptive mechanism, in which the behavioral investment of resources is responsive to environmental cues of risk.

The COM-B model for behavior change cites capability, opportunity, and motivation as the three central components of behavior change [16]. Rather than focusing on an individual’s physical or psychological ability to engage in health behaviors (capability), or the external factors that make certain health behaviors possible (opportunity), the UMRH focusses on motivation as a driver of behavior. This addresses the impact of environmental cues of risk on the unconscious cognitive processes that direct and inspire health behaviors. Therefore, socioeconomic differences in health behaviors may reflect differences in motivation to invest in health effort, rather than constraints in an individual’s ability to look after their health. We suggest that these differences in motivation arise in response to differences in levels of exposure to uncontrollable mortality risk.

In sum, the UMRH argues that environmental cues of risk contribute to the shaping of health behaviors, and that psychological mechanisms respond to the presence of these environmental cues to determine one’s optimal level of investment in preventative health. The extent to which an individual has *objective* control over the mortality risks they face will impact their *perceived* sense of control. The level of PUMR, cued by environmental risk factors, then drives individual investment in health. This is the basis for the UMRH: people who face greater risk of death from factors that are beyond their control are expected to be less motivated to invest in preventative health behaviors [1].

### Measuring PUMR

**Fig. 1.**
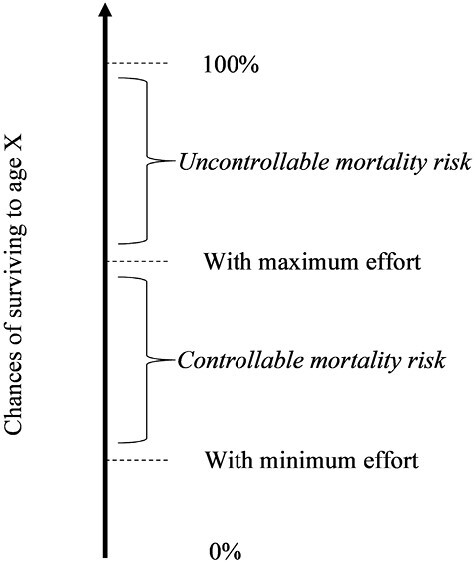
A schematic diagram showing measures of perceived uncontrollable and controllable mortality risk. *Note*: This schematic is taken from Pepper and Nettle [17] in which uncontrollable and controllable mortality risk were defined as extrinsic and intrinsic mortality risk, respectively. The perceived uncontrollable mortality risk is the difference between 100% and the perceived chances of surviving to the mean life expectancy for the population of interest with maximum effort in looking after health. It is the portion of mortality risk that the individual believes they cannot reduce via health effort. The perceived controllable mortality risk is the difference between the perceived chances of living to this age with maximum effort in looking after health, and with minimum effort in looking after health. It is the portion of perceived mortality risk which the individual believes they can reduce via health effort.

Pepper and Nettle [17] introduced a novel approach to measuring PUMR by asking “*If you made the maximum effort you could make to look after your health and ensure your safety, what do you think the chances would be that you would live to be X or more? 0 is ‘no chance’ and 100 is ‘definitely*.*’*” X should be replaced by the mean life expectancy for the population of interest. PUMR is calculated by subtracting the response to this question from 100. Participants are then asked “*If you made no effort at all to look after your health and ensure your safety, what do you think the chances would be that you would live to be X or more? Again, 0 is ‘no chance’ and 100 is ‘definitely.’*” The level of perceived controllable mortality risk (that portion of mortality risk that the individual believes can be mitigated by health effort) is provided by calculating the difference between the answer to the first and second question (see Fig. 1).

### Research Investigating PUMR

In their article, *“Perceived extrinsic mortality risk and reported effort in looking after health: testing a behavioral ecological prediction*,” Pepper and Nettle [17] used the measure of PUMR described above to empirically test the behavioral ecological prediction that environmental cues of uncontrollable risk of death should predict lower investment in preventative health behavior. Their online survey of North American adults found that subjective socioeconomic position was a significant predictor of reported health effort, controlling for age, sex, and income (an association that is commonly reported in public health literature [18]). However, this relationship was weakened, and no longer significant, when PUMR was added to their model as a predictor of health effort (approximately 90% of the total effect of subjective socioeconomic position on health behavior was mediated by PUMR; see Supplementary Table S1). Additionally, higher PUMR was strongly associated with lower reported health effort, providing further support for the claim that PUMR may be key to explaining socioeconomic gradients in preventative health behaviors.

Pepper and Nettle [1] also experimentally primed PUMR in multiple studies by providing participants with manipulated area-level life expectancy projections and stating whether these were or were not caused by controllable individual behaviors. They found that priming PUMR influenced a subsequent health decision—that of choosing a healthy food reward versus an unhealthy alternative. This provided further support to the notion that people who perceive that uncontrollable mortality risk is heightened, will be less likely to make a healthy choice when presented with an unhealthy alternative. Additionally, we used the measure described above to investigate levels of PUMR during the first UK lockdown in response to the COVID-19 pandemic [19, 20]. Levels of PUMR were higher when taking the threat of COVID-19 into account and were associated with lower reported adherence to Government advice on diet and physical activity, and higher levels of smoking.

More recently, an online survey of a nationally representative sample of 1,500 UK adults investigated the relationships between PUMR, health effort, and perceptions of control over specific causes of death [21, 22]. The findings supported the previous negative relationship between PUMR and health effort. We also found that perceptions of control over specific causes of death (except for cardiovascular disease) did not predict overall PUMR, suggesting that overall perceptions of control over mortality risk are not largely driven by perceptions of control over specific causes of death. Instead, PUMR was predicted by variables closely related to exposure to risk and resource availability (perceived neighborhood safety and income). We suggested that PUMR may capture people’s “general sense” of mortality risk, influenced by both exposure to hazards and the availability of resources to avoid threats to health and longevity. However, we emphasized the need for further research to explore other drivers of PUMR and to investigate the accuracy of perceptions of mortality risk. Finally, Isch et al. [23] recently conducted a longitudinal study of risk perceptions in the USA, the findings from which offer support for the association between PUMR and objective measures of risk, using data from the Global Burden of Diseases, Injuries, and Risk Factors Study [24]. Isch et al. [23] recruited an online sample of US participants and found that uncontrollable risks (as indexed by the environmental/occupational summary exposure variable from the Global Burden of Diseases project) significantly predicted PUMR. This suggests that levels of PUMR are related to personalized objective measures of environmental risk.

The findings discussed above offer initial support for the predictions of the UMRH: that people who face greater risk of death from factors that are beyond their control should be less motivated to invest in preventative health behaviors [1]. Despite this evidence, there is a need to replicate and extend the original work of Pepper and Nettle [17]. A meta-analytic summary of the existing data will further allow us to estimate the central effect size for the relationship between PUMR and health effort across studies. There is also a need to evaluate and compare this measure of PUMR with related measures in the literature to better understand the role of PUMR in explaining socioeconomic differences in health behaviors.

Much of the previous literature investigating the relationship between perceptions of control and health behaviors have focused on the construct of health locus of control [25–28]. Health locus of control refers to the extent to which individuals attribute the condition of their health to factors that are either within or beyond their personal control [25–27]. The original version of the Multidimensional Health Locus of Control Scale (MHLC; forms A and B) consists of three six-item dimensions: internality, powerful others, and chance externality [25, 26]. A recent systematic review of studies investigating the relationship between health locus of control and health behaviors found that people who have an internal locus of control were more likely to engage in positive health behaviors, whereas external locus of control was associated with negative health behaviors [29]. Of these dimensions, we would expect that the effects of the internality dimension would be comparable to those of perceived controllable mortality risk, while the chance externality dimension would be comparable to PUMR. By comparing the impact of PUMR on health effort with that of dimensions of health locus of control, we can assess the strength of the relationship between PUMR and health effort in relation to a closely related concept. This analysis provides valuable insights into the extent to which PUMR influences health effort compared with the effects of health locus of control, which have been extensively documented.

The aims of this study are fourfold: (a) conduct a replication of Pepper and Nettle (2014); (b) investigate the effect of choosing different longevity statistics as anchors when measuring PUMR; (c) compare the influence of PUMR on health effort with that of a related measure, the MHLC Scale; and (d) conduct a meta-analysis to evaluate the overall relationship between PUMR and health effort.

## Methods

This study was approved by the Northumbria University research ethics system (ethical approval number 4621). We conducted a replication of Pepper and Nettle [17], and a secondary analysis of their original data comparing the predictive effect of PUMR on health effort with that of dimensions from the MHLC scale. We also compared the use of different longevity statistics used to capture PUMR and conducted a mini meta-analysis of the overall relationship between PUMR and health effort.

### Replication of Pepper and Nettle (2014)

Pepper and Nettle (2014) used three regression models to examine the mediation by PUMR of the relationship between subjective socioeconomic position and self-reported health effort. Model 1 used subjective socioeconomic position as a predictor of health effort. Model 2 used subjective socioeconomic position as a predictor of PUMR. Model 3 included both subjective socioeconomic position and PUMR as predictors of self-reported health effort. Age, sex, and income were controlled for in all models. The statistical significance of mediation was examined using a Sobel test (to which we added our calculation of the proportion of effect mediated; see Supplementary Table S1 for full results). We conducted an exact replication of Pepper and Nettle (2014) using data from Brown et al. (2023), following the analysis protocol of the original study. To provide an exact replication requires the researchers to follow as precisely as possible the procedures used in the original study [30]. The data from Brown et al. (2023) captured PUMR using the same measures of PUMR and health effort as Pepper and Nettle (2014). PUMR was captured using the measure described above, and health effort was measured by asking, “*How much effort do you make to look after your health and ensure your safety these days?*” on a scale from 0 “*no effort at all*” to 100 “*the maximum effort you could make.*” The UMRH posits that uncontrollable mortality risk should influence overall levels of investment in preventative health. Therefore, much of the research investigating the effects of PUMR have used the general measure of health effort described above, rather than using measures of specific health behaviors. Subjective discretionary income was used as a proxy for subjective socioeconomic position. Subjective discretionary income was captured using a previously validated scale from O’Guinn and Wells [31, 32]. This scale is a good indicator of subjective socioeconomic positions as it asks participants to respond to three statements about the degree to which their household finances can satisfy their wants and needs on a five-point Likert scale ranging from “strongly disagree” to “strongly agree.” Using data from Brown et al. (2023), the final sample size for this replication (*n* = 1463) was over three times larger than that of the original analysis from Pepper and Nettle (2014; *n* = 438).

### PUMR Scores Using Different Measures of Longevity

When conducting psychological research that involves asking individuals about their perceived likelihood of living to a given age, the choice of life expectancy statistic has implications for the interpretations of the findings. Two common measures used in research are the mean life expectancy at birth and the median age at death. Mean life expectancy at birth refers to the average number of years that a newborn may be expected to live, given the death rates prevailing at the time of birth [33]. Median age at death captures the age before which half of all deaths occur [34]. Recent research measuring PUMR has used mean life expectancy at birth as the age reference when asking participants to indicate their perceived likelihood of living to a certain age given their maximum effort to look after their health [19–23]. However, a single measure of longevity may fail to fully characterize the mortality situation of a target population [35]. For example, it is common for people to misinterpret an increase in life expectancy at birth as an increase in the age at which most people die [36]. However, increases in the projected average age at death might not be uniform across all age brackets in a population, and may be influenced more by a reduction in infant deaths than an extension to the lifespan. Arguably, using median age at death as an anchor could provide individuals with a reference that is more easily interpreted because it represents the age at which a significant portion of the population has died. On the other hand, mean life expectancy at birth offers a broader perspective, reflecting the average number of years a person born in a particular year is expected to live, encompassing trends, and improvements in healthcare and societal conditions. Therefore, it is important to investigate the influence that choice of life expectancy statistics has on perceptions of uncontrollable mortality risk and its relationship with health effort. Using data from Brown et al. (2023), we investigated the impact of using different age anchors when measuring PUMR. Participants provided two scores for PUMR in response to gender-specific age anchors. Firstly, PUMR was measured using the Office for National Statistics’ figures for life expectancy at birth in the UK (male = 79, female = 83, and those who reported belonging to another gender were shown the population average = 81) and secondly, using the median age at death in the UK (male = 82, female = 86, overall population = 84; [34]).

**Fig. 2.**
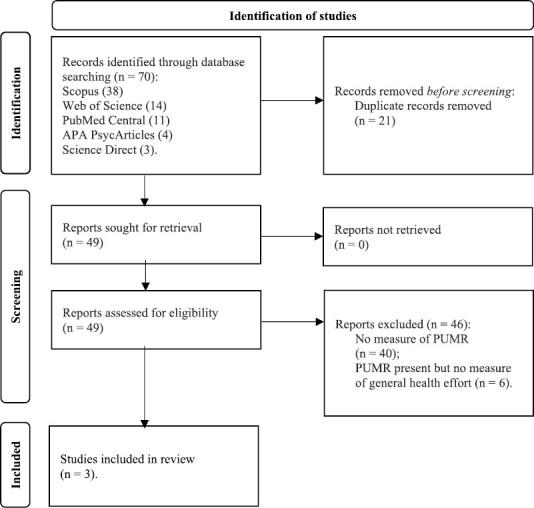
PRISMA flow diagram for selection of studies for meta-analysis. *Note*. The inclusion criteria were that the studies must contain both a measure of Perceived Uncontrollable Mortality Risk (PUMR; previously Perceived Extrinsic Mortality Risk) and a measure of general self-reported health effort (see methods section). The format for this flow diagram is from: Page MJ, McKenzie JE, Bossuyt PM, Boutron I, Hoffmann TC, Mulrow CD, et al. The PRISMA 2020 statement: an updated guideline for reporting systematic reviews. BMJ 2021;372:n71. doi: 10.1136/bmj.n71.

### Comparing PUMR and the MHLC Scale

Using data collected for the study reported in Pepper and Nettle [17], we compared participant PUMR scores with the extensively used, and related measure, the MHLC scale. By capturing perceptions of individual control relevant to a health outcome (risk of death), comparisons can be drawn between PUMR and health locus of control: a construct that aims to capture the extent to which someone’s health is believed to be under their control [25–27]. The original version of the MHLC scale (forms A and B) consists of three six-item dimensions: internality, powerful others, and chance externality [25, 26]. Previous research has suggested that MHLC-chance externality may reflect beliefs of control over a range of environmental factors [37, 38]. We conducted correlational analyses between PUMR, perceived controllable mortality risk, and the MHLC chance and internal dimensions. We ran a multiple regression model to compare the predicted effect of PUMR on health effort with the relevant effects of these two dimensions of the MHLC scale.

### Mini Meta-analysis

A mini meta-analysis is a meta-analytic summary of a small number of studies, usually of researchers’ own studies, presented within a single manuscript. Researchers have called for greater use of mini meta-analyses to help redirect attention toward effect sizes and away from individual studies’ *p*-values [39]. A meta-analytic summary of the findings obtained from a small group of studies provides a more persuasive assessment than considering each effect individually [39, 40]. We meta-analyzed data from the three available studies that capture both PUMR and self-reported health effort, using a random-effects meta-analysis model implemented using the R package “metaphor” [41]. We investigated the overall relationship between PUMR and health effort using Pearson’s correlation coefficient with Fisher’s *z*-transformation as the measure of effect size. The overall relationship between PUMR and health effort was considered significant if the 95% confidence interval of the pooled effect did not include zero. Because this measure of PUMR has not yet been extensively used in the literature, a mini meta-analysis assessing the magnitude of effect from the available studies will provide an initial indication of how meaningful the relationship is and whether further study is warranted.

### Data

#### Replication

For our replication of Pepper and Nettle, we used data from Brown et al. (2023), the findings from which are published in “*Individual characteristics associated with perceptions of control over mortality risk and determinants of health effort*” and “*Perceptions of control over different causes of death and the accuracy of risk estimations*” [21, 22]. For these data, we recruited a nationally representative online sample of 1,500 UK participants via Prolific [prolific.com] (see Table 1 for sample characteristics).

**Table 1:** Available Datasets Measuring Both Perceived Uncontrollable Mortality Risk (PUMR) and Health Effort

	Pepper and Nettle (2014)	Brown et al. (2023)	Isch et al. (2023)
Country	USA	UK	USA
Data collection	July 2012	May 2022	March, May, July, and October 2021
Sample size	521	1463	325
Age	Mean = 28.41 *SD* = 9.71 Range = 18–72	Mean = 45.57 *SD* = 15.53 Range = 18–87	Mean = 52.95 *SD* = 13.74 Range = 19–77
Gender	Man = 216 (41.46%) Woman= 303 (58.16%) Not specified = 2 (0.38%)	Man = 714 (48.80%) Woman= 742 (50.72%) Different gender identity = 7 (0.48%)	Man = 147 (45.23%) Woman = 177 (54.46%) Different gender identity = 1 (0.31%)
Ethnicity	Not available	White = 1276 (87.22%) Asian = 107 (7.31%) Black = 42 (2.87%) Mixed race = 22 (1.50%) Other (not specified) = 16 (1.09%)	Recruited via Prolific, providing a sample that approximately mirrors the ethnic distribution of the US population at the time of data collection.
Socioeconomic factors	*Personal annual income* Mean = $38,360.94 *SD* = $75,272.17 Range = $0–1,500,000 *Subjective socioeconomic* *position (min = 3, max = 21)* Mean = 11.18 *SD* = 4.93 Range = 3–21	*Monthly discretionary income* Mean = £306.31 *SD* = £463.63 Range = −£1,100–7,500 (negative values represent greater outgoings than incomings) *Subjective Discretionary Income* *(min* = 3, *max* = 15) Mean = 8.50 *SD* = 2.64 Range = 3–15 *Years in post-16 education* Mean = 5.12 *SD* = 3.48 Range = 0–23	*Annual family income* Under $15,000 = 29 (8.92%) $15,001–25,000 = 32 (9.85%) $25,001–35,000 = 28 (8.62%) $35,001–50,000 = 45 (13.85%) $50,001–75,000 = 59 (18.15%) $75,001–100,000 = 41 (12.62%) $100,001–150,000 = 23 (7.08%) $150,000–200,000 = 12 (3.69%) Over $200,000 = 7 (2.15%) *Education* Less that High School= 1 (0.31%) High School = 35 (10.77%) Some College = 70 (21.54%) 2-year degree = 35 (10.77%) 4-year degree = 121 (37.23%) Master’s degree = 51(15.69%) Doctoral degree = 12(3.69%)
Perceived uncontrollable mortality risk	Mean = 28.37 *SD* = 30.69 Range = 0–100	Mean = 26.91 *SD* = 17.08 Range = 0-100	Mean = 25.51 *SD* = 22.61 Range = 0-100
Health effort	Mean = 56.25 *SD* = 27.15 Range = 0–100	Mean = 67.43 *SD* = 19.62 Range = 0–100	Mean = 71.90 *SD* = 22.81 Range = 0–100
Correlation coefficient^a^*	*r =* −0.66 *p* < .01	*r =* −0.25 *p* < .01	*r =* −0.15 *p* < .01

*Note. N* = the number of participants for each study that provided scores for both PUMR and Health Effort.

^a^Correlation coefficient represents the Pearson r value for the correlation between PUMR and self-reported health effort for each study.

#### Secondary analysis

For our secondary analysis, we used the original data from Pepper and Nettle (2014), the findings from which are published in “*Perceived Extrinsic Mortality Risk and Reported Effort in Looking after Health*” [17]. Pepper and Nettle surveyed 600 North American online participants using the SocialSci survey platform [socialsci.com] (see Table 1 for sample characteristics).

#### Mini meta-analysis

For our mini meta-analysis, we used data from the two studies described above, plus data from our recent study, reported in “*Objective risk exposure, perceived uncontrollable mortality risk, and health behaviors”* [23]. Data were gathered from a USA-representative longitudinal survey study of 915 participants recruited via Prolific (see Table 1 for final sample characteristics once exclusions and missing data were accounted for). Additionally, we conducted a literature search to determine whether any further data should be included in our mini meta-analysis of the relationship between PUMR and health effort (see Fig. 2). We searched five databases, including Scopus, Web of Science, PubMed Central, APA PsycArticles, and Science Direct. We applied the following criteria to filter search results: (a) Publications had to be in English, with no geographic restrictions on publication; (b) Only quantitative studies were included; (c) Studies had to include a measure of PUMR, which was searched using terms “perceived uncontrollable mortality risk,” “perceptions of uncontrollable mortality risk,” and “perceived extrinsic mortality risk”; and (d) Studies had to include the general measure of investment in health effort as described in Pepper and Nettle (2014) (which is described in the section *“Replication of Pepper and Nettle (2014)”* above), using search terms “health effort,” “preventative health behavior,” and “investment in health.” All data were searched, scanned, and screened for eligibility by one researcher, before full discussion and agreement among the full research team. The search yielded the same three studies as previously described, confirming that our selected studies represent the available data for the relationship between PUMR and health effort.

While the three studies included in this article are cross-sectional and, as such, cannot establish causality, we employ causal language (e.g., “predictors” and “mediators”) to reflect the theoretical framework of the UMRH. Our approach allows us to investigate potential causal pathways between perceptions of uncontrollable mortality risk and health behaviors. Previous experimental work (described in the section “*Research investigating Perceived Uncontrollable Mortality Risk*” above) has shown that priming PUMR can causally effect health choices [1], however, we acknowledge the need for caution when interpreting the associations included in our meta-analysis.

### Analysis

All statistical analyses were performed using R [42]. The following packages were used for data processing, analysis, and visualization: apaTables [43], bestNormalize [44], dplyr [45], GGally [46], ggcorrplot [47], ggiraphExtra [48], ggplot2 [49], ggpubr [50], ggrepel [51], gridExtra [52], jmv [53], lsr [54], metafor [41], multilevel [55], plotly [56], psych [57], and tidyverse [58]. Our full R script is available on the Open Science Framework (OSF; osf.io/dgwna).

## Results

### Replication of Pepper and Nettle (2014)

In a linear regression model, PUMR predicted lower reported health effort when controlling for age, gender, discretionary income, and subjective discretionary income (*F*_5,1450_ = 111.54, *p* < .001, β = −0.23, SE [β] = 0.03). Subjective discretionary income (used as a proxy for socioeconomic position) predicted health effort when controlling for age, gender, and discretionary income (*F*_4,1451_ = 56.30, *p* < .001, β = 0.17, SE [β] = 0.03). This relationship was weakened when PUMR was added to the model (*F*_5,1450_ = 33.68, *p* < .001, β = 0.13, SE [β] = 0.03) and PUMR mediated 24% of the effect of subjective discretionary income on health effort (95% CI [0.15, 0.38], *p* < .001), supported by a significant Sobel test (*z* = 5.79, *p* < .001; see Table 2 for full results). Despite reporting smaller effect sizes than the original study (see Table 2, and Supplementary Table S1 for the original findings from Pepper and Nettle (2014)), this result provides a successful replication of Pepper and Nettle [17].

**Table 2:** A Summary of the Models Used to Examine the Mediation by PUMR of the Relationship Between Subjective Discretionary Income and Reported Health Effort

		β	Standard error [β]	*F* ratio	*p*	Lower (95% CI)	Upper (95% CI)	*ηp*2
Model 1	Subjective discretionary income as a predictor of health effort	0.17	0.03	56.30	<.001	0.11	0.23	.02
Model 2	Subjective discretionary income as a predictor of PUMR	−0.15	0.03	55.07	<.001	−0.10	−0.21	.02
Model 3	PUMR as a predictor of health effort with subjective discretionary income controlled	−0.23	0.03	111.54	<.001	−0.18	−0.28	.05
	Subjective discretionary income as a predictor of health effort with PUMR controlled	0.13	0.03	33.68	<.001	0.08	0.19	.01
Mediation	Sobel *Z* = 5.79, *p* < .001 Proportion mediated = 0.24 (95% CI [0.15, 0.38], *p* < .001)							

*Note.* Age, sex, and discretionary income are also controlled in all models, *n* = 1463 for all models, and β represents standardized regression weights.

### PUMR Scores Using Different Measures of Longevity

**Fig. 3.**
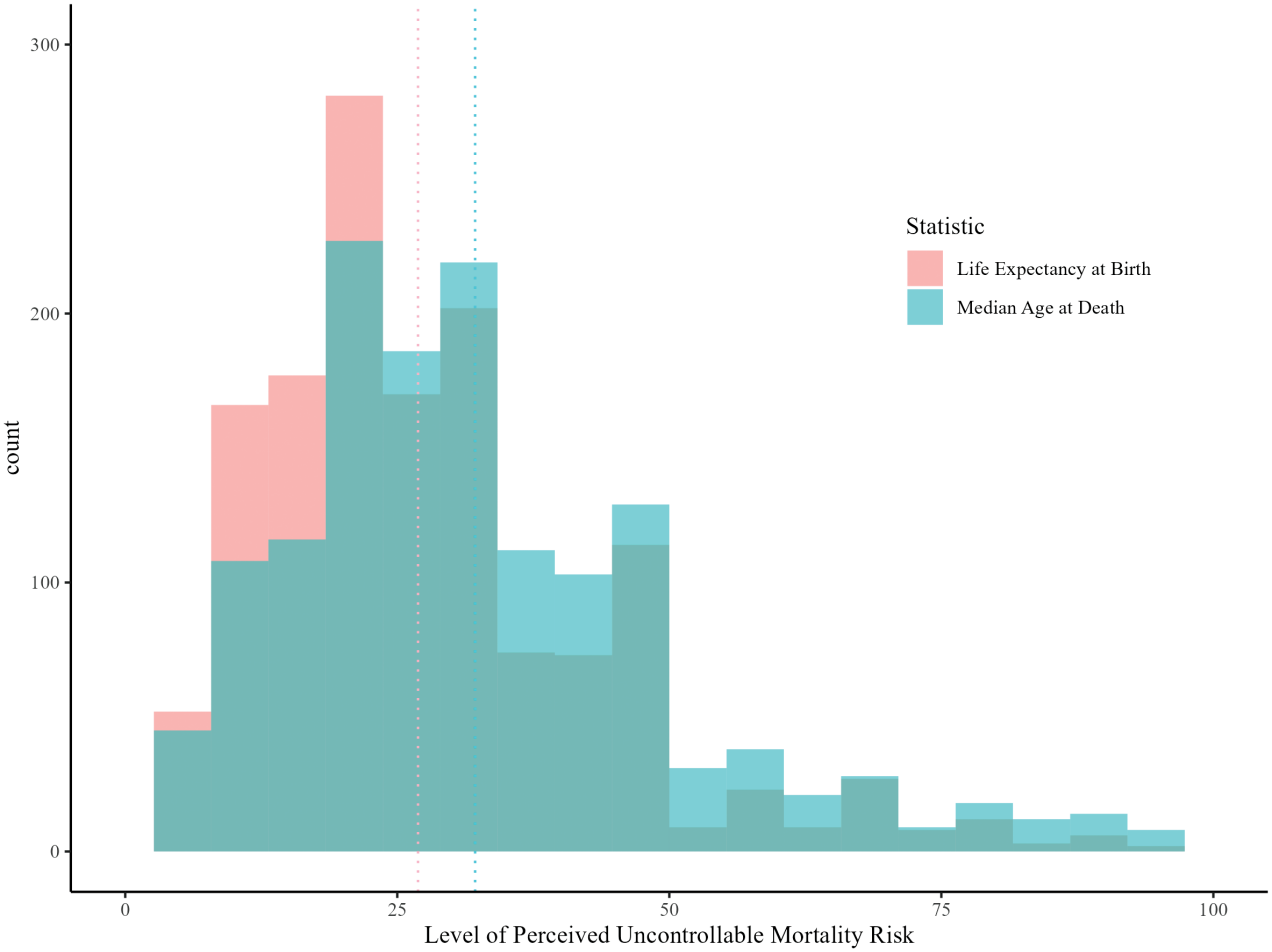
Paired histograms for PUMR scores, comparing mean life expectancy at birth and median age at death statistics. *Note*. Dashed lines represent mean scores for PUMR using each longevity statistic. PUMR with respect to median age at death: *M* = 32.16, *SD* = 19.01. PUMR with respect to mean life expectancy at birth: *M* = 26.91, *SD* = 17.08. *N* = 1,463.

Using data from Brown et al. (2023), PUMR scores were higher when captured using median age at death (*M* = 32.16, *SD* = 19.01), compared with mean life expectancy at birth (*M* = 26.91, *SD* = 17.08), reflecting the lower perceived likelihood of living to an older age (see Fig. 3). However, the two measures were strongly correlated (*r* = 0.87, *p* < .001) and both measures of PUMR were associated with lower reported health effort to a similar degree (using mean life expectancy at birth, *r = −*0.25, *p* < .001; using median age at death, *r = −*0.24, *p* < .001).

### Comparing PUMR and Dimensions of the MHLC Scale

We found small significant correlations between the measures of MHLC-internality and perceived controllable mortality risk (*r* = 0.21, *p* < .001), as well as between MHLC-chance externality and PUMR (*r* = 0.15, *p* < .001; see Table 3). In terms of the predictive validity of PUMR compared with MHLC dimensions, a linear regression model found that the two MHLC dimensions explained only 1% of the variance in self-reported health effort (*R*^2^ = 0.01, *F*_2,518_ = 3.41, *p* = .03). In a separate model, the measures of perceived uncontrollable and controllable mortality risk explained 44% of this variance (*R*^2^ = 0.44, *F*_2,518_ = 199.20, *p* < .001). PUMR was the most notable predictor of self-reported health effort when compared with perceived controllable mortality risk and MHLC dimensions (β = −0.71, 95% CIs = −0.63, −0.79). When controlling for perceived controllable mortality risk and MHLC dimensions, PUMR accounted for 31% of the unique variance in self-reported health effort (*sr*^2^ = 0.31, 95% CIs = 0.25, 0.37). For full comparison of perceived uncontrollable and controllable mortality risk variables with MHLC dimensions, see Table 4 and Supplementary Tables S2, S3.

**Table 3:** Means, Standard Deviations, and Correlation for Mortality Risk Perception Variables, MHLC Scores and Self-reported Health Effort (*n* = 572)

Variable	*M*	*SD*	1	2	3	4
1. Perceived uncontrollable mortality risk	28.37	30.69				
2. Perceived controllable mortality risk	34.21	28.46	−0.62**			
			[−0.67, −0.57]			
3. MHLC-Chance	17.97	5.07	0.15**	−0.23**		
			[0.06,0.23]	[−0.31, −0.15]		
4. MHLC-Internal	25.46	4.68	−0.17**	0.21**	−0.31**	
			[−0.25, −0.09]	[0.12, 0.29]	[−0.38, −0.23]	
5. Health effort	56.25	27.15	−0.66**	0.35**	−0.04	0.11**
			[−0.70, −0.60]	[0.28, 0.43]	[−0.13, 0.04]	[0.03, 0.20]

*Note. M* and *SD* are used to represent mean and standard deviation, respectively. Values in square brackets indicate the 95% confidence interval for each correlation. The confidence interval is a plausible range of population correlations that could have caused the sample correlation (Cumming, 2014). * indicates *p* <.05. ** indicates *p* <.01.

**Table 4:** Regression Results Predicting Reported Health Effort from PUMR, Perceived Controllable Mortality Risk, the MHLC Chance Dimension and MHLC-internal Dimension (*n* = 572)

Predictor	*b*	*b* 95% CI [LL, UL]	β	*beta* 95% CI [LL, UL]	*sr* ^*2*^	*sr* ^2^ 95% CI [LL, UL]	*r*	Fit
(Intercept)	68.09**	[53.56, 82.62]						
Perceived uncontrollable mortality risk	−0.63**	[−0.70, −0.55]	−0.71	[−0.79, −0.63]	0.31	[0.25, 0.37]	−0.66**	
Perceived controllable mortality risk	−0.08	[−0.16, 0.00]	−0.08	[−0.17, 0.00]	0.00	[−0.00, 0.01]	0.35**	
MHLC-chance	0.27	[−0.10, 0.65]	0.05	[−0.02, 0.12]	0.00	[−0.00, 0.01]	−0.04	
MHLC-internal	0.15	[−0.26, 0.55]	0.03	[−0.04, 0.09]	0.00	[−0.00, 0.00]	0.11**	
								*R* ^*2*^ = 0.437**
								95% CI [0.37, 0.49]

*Note.* A significant *b*-weight indicates the beta-weight and semi-partial correlation are also significant. *b* represents unstandardized regression weights. *beta* indicates the standardized regression weights. *sr*2 represents the semi-partial correlation squared. *r* represents the zero-order correlation. *LL* and *UL* indicate the lower and upper limits of a confidence interval, respectively. * indicates *p* < .05. ** indicates *p* < .01.

### Mini Meta-analysis

**Fig. 4.**
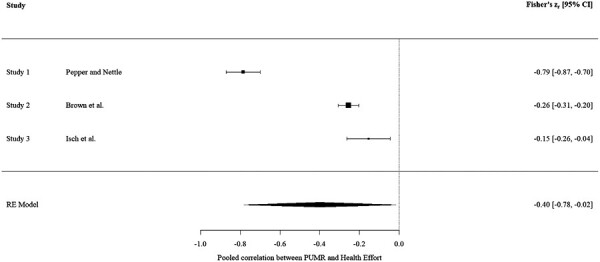
Forest plot showing mini meta-analysis of the relationship between PUMR and Health Effort

Our meta-analysis of the association between PUMR and self-reported health effort from the three available studies showed a significant negative relationship between the two variables of interest with a medium central effect size [59](see Fig. 4).

## Discussion

This study replicated the research conducted by Pepper and Nettle (2014) and provides support for their finding that PUMR mediates the negative association between subjective socioeconomic position and health effort. Our results demonstrate that this measure of PUMR is a much stronger predictor of health effort compared with the internal and chance dimensions of the MHLC scale. Additionally, a mini meta-analysis of the available research demonstrates a significant negative relationship between PUMR and health effort. Overall, these findings lend support to the UMRH, which suggests that people who experience greater uncontrollable mortality risk should be less motivated to invest in preventive health behaviors. We discuss several areas for future study to further explore the relationship between PUMR and health effort and to examine the implications of the UMRH.

Firstly, as with Pepper and Nettle (2014), our analysis of the data from Brown et al. (2023) found that PUMR predicted lower reported health effort when controlling for age, gender, and income. Additionally, subjective discretionary income (used as a proxy for socioeconomic position) predicted health effort when controlling for age, gender, and income. This relationship was weakened when PUMR was added to the model, and offers support for the original findings of Pepper and Nettle (2014). However, PUMR mediated approximately a quarter of the relationship between subjective discretionary income and health effort, compared with mediating 90% of the total effect of subjective socioeconomic position on health effort using Pepper and Nettle’s original data. Furthermore, the correlation coefficient between PUMR and health effort was markedly weaker when using data from Brown et al. (2023) compared with the findings of Pepper and Nettle (2014). It is not uncommon for replication studies to find weaker effects than the original findings. Theoretical consideration of how to interpret replication results has argued that the strongest original effects can be expected to weaken the most because of “regression to the mean” being an inevitable property of replication research [60]. Stanley and Spence [61] used computer-generated simulations of replication results to test the impact of measurement error on replication attempts. They found that effect size estimates from a published study can deviate substantially from the true effect size simply due to measurement error, suggesting that researchers should move away from viewing replication attempts as verifying or falsifying published findings, and toward a meta-analytic mind-set. This emphasizes the importance of further exploring replication study findings by meta-analyzing the relationship between variables of interest [30]. A mini meta-analysis serves as a useful technique for quantifying the overall strength of a relationship. Our mini meta-analysis of the association between PUMR and self-reported health effort from the three available studies showed a significant negative relationship between the two variables of interest with a medium effect size [59]. This suggests that the relationship is non-trivial, warranting further study. A previous meta-analysis of 76 independent samples including 76,580 adult participants investigated the relationships between the MHLC scale and specific health behaviors [27]. Though the included studies did not use the same measure of health effort as our current study, measures of specific health behaviors (diet, exercise, smoking, and alcohol consumption) provide a good indication of overall health effort. Averaged Pearson’s *r* values for the relationships between the chance dimension of the MHLC scale (that most comparable to PUMR) and specific health behaviors were weak, ranging from −0.07 to 0.08, and from −0.07 to 0.1 across all three dimensions of the scale. This suggests that PUMR provides a substantially more effective predictor of health effort than the relevant dimensions of the MHLC scale. This offers further support for the UMRH and suggests that those who perceive themselves to be exposed to greater uncontrollable mortality risk, are likely to be less motivated to invest in preventative health behaviors.

Finally, PUMR scores were higher when the measure was anchored by median age at death (male = 82, female = 86, overall UK population = 84), compared with mean life expectancy at birth (male = 79, female = 83, overall UK population = 81). As median age of death is older than mean life expectancy at birth, this difference in PUMR scores most likely reflects the lower perceived likelihood of living to an older age. However, responses to the measure of PUMR using each longevity statistic were strongly correlated, and both were negatively associated with health effort to a similar degree. Therefore, for consistency with previous research, we suggest that researchers should use mean life expectancy at birth when referencing the average expected longevity of target populations in the measure of PUMR [19, 20, 62].

Many questions remain unanswered concerning the relationship between uncontrollable mortality risk and health behavior, as well as regarding the perceptual processes that mediate between the two. Further studies may seek to address some of the following questions to allow the predictions of the UMRH to better inform health campaigns designed to address socioeconomic inequalities in health behaviors.

### Future Areas of Study

#### How accurate is PUMR?

Understanding the extent to which people accurately perceive uncontrollable mortality risk may assist in effectively designing interventions to improve health behaviors. Perceptions of risk can be influenced by a range of factors including personality traits, demographic factors, as well as differences in individual informational environments [63, 64]. As discussed, nascent evidence suggests that PUMR is associated with area-level measures of objective risk [23]. It will be important to understand the extent to which individual perceptions map onto the ecological conditions and prevalence of objectively uncontrollable mortality risk [65]. For example, if an individual accurately perceives their level of uncontrollable mortality risk to be high, they may be less motivated to engage in positive health behaviors. Interventions that encourage people to engage in preventative health behaviors but fail to address the environmental factors that perpetually reinforce those high levels of perceived risk, are likely to be ineffective and could even be deemed unethical. Alternatively, where perceptions of uncontrollable mortality risk do not accurately represent objective measures of risk, interventions that seek to reduce external experiences of risk may be ineffective in fostering positive health behaviors if people do not perceive the safety of their environment to have improved. Understanding the degree to which perceptions of uncontrollable mortality risk map onto ecological conditions may also help to assess the applicability of the UMRH to different populations and environments. It will be important to test the impact of perceptions of uncontrollable mortality risk in environments with high mortality rates and low behavioral control over health. For example, when comparing Tsimane Amerindians in Bolivia with industrialized populations in Japan, the UK, Alami, and Stieglitz [37] found that Tsimane MHLC-Chance scores were negatively associated with the uptake of medical treatment. This is consistent with the predictions of the UMRH, however further research measuring PUMR is needed to test the applicability of this theory to multiple ecological contexts.

#### What informs PUMR?

It is important to determine which cues of mortality risk have the strongest influence over PUMR. For example, are beliefs about controlling people’s own risk of death influenced more by their own family medical history or by rates of violent crime in their area? Understanding which cues of mortality risk carry the most weight will provide insights necessary for delivering interventions to influence health behaviors. Research may look to investigate the influence of potential strong cues of uncontrollable mortality risk to determine whether perceptions drawn from these cues accurately reflect objective levels of risk.

Perceptions of risk can be strongly influenced by a variety of media factors, including the amount of media coverage that a risk receives and the tone in which the information is presented [66]. If the media devote a lot of attention to a specific category of risk, it will become increasingly salient to the public. The news media have been reported to significantly misrepresent the prevalence of leading causes of death which may lead to distorted perceptions of risk among the public [67]. For example, heart disease accounts for close to one-third of all US deaths, yet represents only 2%–3% of media reports on mortality risks [68]. Conversely, violent deaths (including suicide, homicide, and terrorism) account for less than 3% of annual US deaths, yet represent more than two-thirds of media coverage concerning risk of death [68]. As the media landscape diversifies, becomes increasingly online focused, and social media plays a more dominant role, future research must play close attention to the influence that media coverage has on risk perceptions [66]. Given the discussed relationship between PUMR and health behaviors, research into the relationship between online media and risk perceptions may also lead to calls for enhanced journalistic, as well as individual, responsibility to ensure that the way in which information is presented does not adversely affect public health.

#### How aware are people of their own perceptions of uncontrollable mortality risk?

Finally, it is not yet clear whether the relationship between PUMR and health effort is solely dependent on automatic processes or whether it may also involve conscious reflection. For example, Pepper and Nettle [1] found that priming PUMR impacted participants’ choice in food reward but did not influence self-reported health behavior intentions. This suggests that an implicit effect rather than a consciously processed reflection produces behavioral change in response to cues of uncontrollable mortality risk. However, in some cases, reflective processes that consider perceptions of uncontrollable risk may influence people’s behavioral response. For example, Bulley and Henry [69] suggest that employing episodic foresight to consider an uncertain or dangerous environment in one’s future may promote a shortening of time horizons to discount delayed gratification. Further study is needed to understand what is involved in processing cues of uncontrollable mortality risk and how this relates to a wider range of both actual and reported health behaviors.

### Limitations

The present study is not without methodological and theoretical limitations. Firstly, the majority of findings concerning the relationship between PUMR and health behaviors rely on self-reported data. This introduces a degree of subjectivity and potential for response bias, which may obscure the relationship between more objective measures of risk and actual health behaviors. Furthermore, although the UMRH predicts that effects should be universal, the generalizability of existing empirical research and the findings from our mini meta-analysis are limited by sample characteristics (see Table 1). Data were collected from participants in the USA and UK only, thus the cultural, socioeconomic, and demographic characteristics of these regions may influence the relationships between perceptions of risk and health behaviors. The USA and UK are relatively safe countries in global terms [70]. The relationships between PUMR and health effort may differ among citizens of countries that experience much higher levels of risk. The study’s conclusions should be interpreted within the specific sociocultural and geographic boundaries of the samples included. By expanding the scope of data collection to encompass a more globally representative sample, researchers may better understand the potential variations and nuances in the implications of the UMRH. Furthermore, the samples used in this study were recruited via online survey platforms. Research investigating the challenges of collecting data via online survey platforms suggests that users may be more likely to work from home and typically report lower social engagement than the general population [71], which may have implications for experiences of environmental risk [72]. Future research should move beyond previously used online survey platforms to test the generalizability of existing findings. Another potential limitation of our study is that we only compared the relationship between PUMR and health effort against the effects of the MHLC dimensions “internality” and “chance externality,” but not “powerful others.” This was because these selected dimensions were believed to be more readily comparable to perceived controllable mortality risk and PUMR. However, considering the combined effects of all MHLC dimensions on health effort might enable a more comprehensive comparison of PUMR and health locus of control.

### Conclusion

This study replicated Pepper and Nettle (2014), offering support for their original finding that PUMR mediates the negative relationship between subjective socioeconomic position and health effort. We argue that PUMR should be measured using mean life expectancy at birth, in preference to median age at death, to aid the consistent measurement of PUMR. We demonstrate that PUMR is a stronger predictor of health effort than the internal and chance dimensions of the MHLC Scale, which are commonly used in behavioral health psychology. Finally, our mini meta-analysis supports a significant medium-sized negative relationship between PUMR and health effort. Overall, these findings support the UMRH which states that people who experience greater uncontrollable mortality risk should be less motivated to invest in preventative health behaviors. We encourage the expanded use of this measure of PUMR in studies that seek to incorporate evolutionary perspectives in public health. We suggest several areas for future study related to PUMR and its impact on health behaviors. These areas include assessing the accuracy of PUMR and its relationship to objective measures of risk, investigating the cues that inform PUMR and their influence on health behaviors, the role of media in shaping risk perceptions, and the awareness of individuals regarding their own perceptions of uncontrollable mortality risk. Further research is needed to understand these areas and their implications for designing effective interventions and addressing socioeconomic inequalities in health behaviors.

## Supplementary Material

kaad072_suppl_Supplementary_Tables_S1-S3

## Transparency Statements

1. **Study Registration.** This study was not formally registered.
2. **Analytic plan registration.** The analysis plan was not formally preregistered.
3. **Data availability:** De-identified data from this study are available in the following public archives:
  - Data from Brown et al. (2023)—https://osf.io/tvumw/;
  - Data from Pepper and Nettle (2014)—https://static-content.springer.com/esm/art%3A10.1007%2Fs12110-014-9204-5/MediaObjects/12110_2014_9204_MOESM1_ESM.xls; and
  - Data from Isch et al. (2023)—https://github.com/CalvinIsch/pumr-omr.
4. **Analytic code availability.** Analytic code used to conduct the analyses presented in this study are available in a public archive: (OSF; osf.io/dgwna).
5. **Materials availability.** Some of the materials used to conduct the study are presented in a public archive: (OSF; osf.io/dgwna).
