# Supplementary material for: The Relationship Between Perceived Uncontrollable Mortality Risk and Health Effort: Replication, Secondary Analysis, and Mini Meta-analysis
Source: Ann Behav Med. 2024 Jan 8;58(3):192–204. doi: 10.1093/abm/kaad072 (PMC10858306; doi:10.1093/abm/kaad072)
Supplement: kaad072_suppl_Supplementary_Tables_S1-S3 [file kaad072_suppl_supplementary_tables_s1-s3.docx]

**SUPPLEMENT**

**The relationship between perceived uncontrollable mortality risk and health effort: replication, secondary analysis, and mini meta-analysis.**

**Table S1.** A summary of the models used to examine the mediation of the relationship between subjective socioeconomic position and reported effort in looking after health by perceived uncontrollable mortality risk.

From: [Perceived Extrinsic Mortality Risk and Reported Effort in Looking after Health](https://link.springer.com/article/10.1007/s12110-014-9204-5?sa_campaign=email/event/articleAuthor/onlineFirst), Pepper and Nettle (2014)

|  |  | *β* | Standard error [*β*] | *F* ratio | *p* | Lower Bound (95% CI) | Upper Bound (95% CI) | Effect size |
| --- | --- | --- | --- | --- | --- | --- | --- | --- |
| Model 1 | Subjective SEP as a predictor of health effort | 0.56 | 0.28 | 3.94 | 0.048* | 0.01 | 1.11 | 0.009 |
| Model 2 | Subjective SEP as a predictor of perceived extrinsic mortality | –0.83 | 0.31 | 6.97 | 0.009* | –1.45 | –0.21 | 0.016 |
| Model 3 | Perceived extrinsic mortality as a predictor of health effort with Subjective SEP controlled | –0.64 | 0.04 | 244.13 | 0.000* | –0.72 | –0.56 | 0.362 |
|  | Subjective SEP as a predictor of health effort with extrinsic mortality controlled | 0.05 | 0.21 | 0.06 | 0.803 | –0.37 | 0.47 | 0.000 |
| Mediation | Sobel Z = 2.65, *p* = 0.008* |  |  |  |  |  |  |  |
|  | Proportion mediated = 0.90 (95% CI [0.33, 3.56], *p* < 0.03*) | | | | | | |  |

*^Note.^* ^Age, sex, and income are also controlled in all models, df = 1, error = 433, *^*^p^*^< 0.05, effect size = ηp 2. In Pepper and Nettle (2014), PUMR is referred to as perceived extrinsic mortality risk, though is captured using the same measure as PUMR described in this paper.^

**Table S2.** Regression results predicting reported health effort from perceived uncontrollable mortality risk and perceived controllable mortality risk (n=521)

| Predictor | *b* | *b*  95% CI  [LL, UL] | *beta* | *beta*  95% CI  [LL, UL] | *sr^2^* | *sr^2^*  95% CI  [LL, UL] | *r* | Fit |  |
| --- | --- | --- | --- | --- | --- | --- | --- | --- | --- |
| (Intercept) | | 77.01** | [72.35, 81.67] |  |  |  |  |  |  |
| Perceived uncontrollable mortality risk | | -0.63** | [-0.70, -0.56] | -0.71 | [-0.79, -0.63] | .31 | [.25, .37] | -.66** |  |
| Perceived controllable mortality risk | | -0.09* | [-0.16, -0.01] | -0.09 | [-0.17, -0.01] | .00 | [-.00, .01] | .35** |  |
|  | |  |  |  |  |  |  |  | *R^2^*  = .435** |
|  | |  |  |  |  |  |  |  | 95% CI[.37,.49] |
|  | |  |  |  |  |  |  |  |  |

*^Note.^* ^A significant^ *^b^*^-weight indicates the beta-weight and semi-partial correlation are also significant.^ *^b^* ^represents unstandardized regression weights.^ *^beta^* ^indicates the standardized regression weights.^ *^sr2^* ^represents the semi-partial correlation squared.^ *^r^* ^represents the zero-order correlation.^ *^LL^* ^and^ *^UL^* ^indicate the lower and upper limits of a confidence interval, respectively.
* indicates^ *^p^* ^< .05. ** indicates^ *^p^* ^< .01.^

**Table S3.** Regression results predicting reported health effort from MHLC chance and internal dimensions (n=521)

| Predictor | *b* | *b*  95% CI  [LL, UL] | *beta* | *beta*  95% CI  [LL, UL] | *sr^2^* | *sr^2^*  95% CI  [LL, UL] | *r* | Fit |
| --- | --- | --- | --- | --- | --- | --- | --- | --- |
| (Intercept) | 40.45** | [22.32, 58.57] |  |  |  |  |  |  |
| MHLC-Chance | -0.04 | [-0.52, 0.44] | -0.01 | [-0.10, 0.08] | .00 | [-.00, .00] | -.04 |  |
| MHLC-Internal | 0.65* | [0.12, 1.17] | 0.11 | [0.02, 0.20] | .01 | [-.01, .03] | .11** |  |
|  |  |  |  |  |  |  |  | *R^2^*  = .013* |
|  |  |  |  |  |  |  |  | 95% CI[.00,.04] |
|  |  |  |  |  |  |  |  |  |

*^Note.^* ^A significant^ *^b^*^-weight indicates the beta-weight and semi-partial correlation are also significant.^ *^b^* ^represents unstandardized regression weights.^ *^beta^* ^indicates the standardized regression weights.^ *^sr2^* ^represents the semi-partial correlation squared.^ *^r^* ^represents the zero-order correlation.^ *^LL^* ^and^ *^UL^* ^indicate the lower and upper limits of a confidence interval, respectively.
* indicates^ *^p^* ^< .05. ** indicates^ *^p^* ^< .01.^
